# Supplementary material for: Structure Elucidation, Biosynthesis and Biological Evaluation of Neosorangicin A, a Member of the Sorangicin Family
Source: J Nat Prod. 2026 Mar 19;89(4):1226–37. doi: 10.1021/acs.jnatprod.6c00056 (PMC13122636; doi:10.1021/acs.jnatprod.6c00056)
Supplement: Supplementary file 1 [file np6c00056_si_001.pdf]

Supporting Information

Structure Elucidation, Biosynthesis and Biological Evaluation of Neosorangicin A, a Member of the Sorangicin Family

Franziska Fries[1,2,†], Sebastian Walesch[1,†], Rolf Jansen[3], Kristin von Peinen[3], Luisa Mehr[3], Linda Pätzold[4], Sabrina Karwehl[3], Andreas M. Kany[1,2], Ronald Garcia[1,2], Silke Reinecke[3], Jörg Haupenthal[1,2], Theresia E. B. Stradal[3], Markus Bischoff[1,2,4], Marc Stadler[2,3], Rolf Müller[1,2,*], Jennifer Herrmann[1,2,*]

[1]Helmholtz Institute for Pharmaceutical Research Saarland (HIPS), Helmholtz Centre for Infection Research (HZI) and Department of Pharmacy, Saarland University, Campus E8 1, 66123 Saarbrücken, Germany

[2]German Center for Infection Research (DZIF), Partner Site Hannover-Braunschweig, 38124 Braunschweig, Germany

[3]Helmholtz Centre for Infection Research (HZI), Inhoffenstrasse 7, 38124 Braunschweig, Germany

[4]Institute of Medical Microbiology and Hygiene, Saarland University, 66421 Homburg/Saar, Germany

[†]These authors contributed equally.

*Corresponding authors: Rolf Müller, Email: rolf.mueller@helmholtz-hips.de and Jennifer Herrmann, Email: jennifer.herrmann@helmholtz-hips.de.

Table of Contents

Supplementary Tables 3

Table S1. 3
Table S2. 5
Table S3. 6
Table S4. 7
Table S5. 9
Table S6. 10
Table S7. 11

Supplementary Figures 13

Figure S1. 13
Figure S2. 13
Figure S3. 13
Figure S4. 14
Figure S5. 15
Figure S6. 16
Figure S7. 17
Figure S8. 18
Figure S9. 19
Figure S10. 20
Figure S11. 21
Figure S12. 22
Figure S13. 23
Figure S14. 24
Figure S15. 25
Figure S16. 26
Figure S17. 27
Figure S18. 28
Figure S19. 29

References 30

Supplementary Tables

Table S1. NMR spectroscopic data of neosorangicin A (**1**) acquired at 700/175 MHz in methanol-d_4.

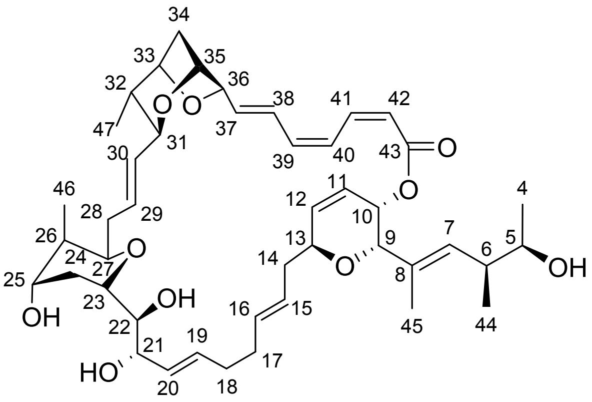

Position	δ_C[a] [ppm], type	δ_H[b] [ppm], mult	COSY[c]	ROESY[d]	HMBC[e]
4	19.3, CH3	1.01, d (6.5)	5	(s)[f] 5, 6, 7, 44; (w)[g] 27, 29	5, 6
5	71.8, CH	3.56, qd (6.3, 6.3, 6.3, 4.5)	4, 6	(s) 4, 6, 7, 44; (w) 45	4, 6, 7, 44
6	40.3, CH	2.53, dqd (9.9, 6.9, 6.9, 6.9, 4.5)	5, 7, 44	(s) 4, 5, 7, 44, 45	4, 5, 7, 8, 44
7	130.0, CH	5.39, dquin (9.9, 1.3, 1.3, 1.3, 1.3)	6, 9, 45	(s) 4, 5, 6, 9, 44; (w) 40	5, 6, 8, 9, 10, 44, 45
8	133.4, C				
9	74.3, CH	4.25, br s	7, 10, 45	(s) 7, 10, 14a, 45; (w) 15	7, 8, 10, 13, 45
10	66.9, CH	5.32, dd (5.8, 1.7)	9, 11	(s) 9, 11, 45	9, 11, 12, 43
11	123.9, CH	6.03, ddd (9.9, 5.8, 2.2)	10, 12, 13	(s) 10, 12	9, 10, 12, 13
12	137.1, CH	6.14, dd (10.0, 3.1)	11, 13	(s) 11, 13, 14b	10, 11, 13
13	75.3, CH	4.41, m	11, 12, 14a, 14b	(s) 12, 14b, 15; (w) 14a	9, 11, 12, 14, 15
14a	35.5, CH2	2.39, ddd (14.0, 10.7, 6.8)	13, 14b, 15	(s) 9, 14b, 16; (w) 13	12, 13, 15, 16
14b		2.15, m	13, 14a, 15	(s) 12, 13, 14a, 16	13, 15
15	128.5, CH	5.53, m	14a, 14b	(s) 13, 17a, 17b; (w) 9	14, 16
16	134.0, CH	5.53, m	17a, 17b	(s) 14a, 14b, 18a, 18b	15, 17, 18
17a	33.9, CH2	2.15, m	16, 18b	(s) 15, 19	15, 16, 18, 19
17b		2.08, m	16, 18a	(s) 15, 18a, 19	15, 16, 18, 19
18a	34.7, CH2	2.16, m	17b, 19	(s) 16, 17b, 20	16, 17, 19, 20
18b		2.08, m	17a, 19	(s) 16, 20	16, 17, 19, 20
19	134.6, CH	5.72, ddd (15.0, 8.0, 5.6)	18a, 18b, 20	(s) 17a, 17b, 21	18, 21
20	129.9, CH	5.58, dd (15.4, 7.9)	19, 21	(s) 18a, 18b, 21, 23	18, 19, 21, 22
21	74.5, CH	4.16, dd (7.5, 4.3)	20, 22	(s) 19, 20, 22; (w) 23, 24a	19, 20, 22, 23
22	77.6, CH	3.47, dd (7.7, 4.3)	21, 23	(s) 21, 24b; (w) 23	20, 21, 23, 24
23	75.1, CH	3.64, ddd (11.6, 7.7, 2.4)	22, 24a, 24b	(s) 20, 24a, 27; (w) 21, 22, 24b	21, 22, 25, 27
24a	31.1, CH2	1.73, m	23, 24b, 25	(s) 23, 24b, 25; (w) 21	25, 26
24b		1.64, ddd (14.6, 11.6, 3.0)	23, 24a, 25	(s) 22, 24a, 25, 46; (w) 23	22, 23, 26
25	71.2, CH	3.85, m	24a, 24b, 26	(s) 24a, 24b, 26, 46	23, 26, 27, 46
26	38.3, CH	1.55, m	25, 27, 46	(s) 25, 27, 46; (w) 28b, 47	24, 25, 46
27	74.8, CH	3.83, m	26, 28a, 28b	(s) 23, 26, 28a, 28b, 29; (w) 4	23, 25, 28, 29, 46
28a	37.2, CH2	2.25, dddd (13.9, 5.6, 4.3, 1.7)	27, 28b, 29	(s) 27, 28b, 29, 46	26, 27, 29, 30
28b		2.16, m	27, 28a, 29	(s) 27, 28a, 30, 46; (w) 26	26, 27, 29, 30
29	133.0, CH	5.45, ddd (15.0, 10.1, 4.1)	28a, 28b, 30	(s) 27, 28a, 31; (w) 4, 47	27, 28, 30, 31
30	133.0, CH	5.37, ddd (15.2, 8.5, 1.8)	29, 31	(s) 28b, 31, 32; (w) 46, 47	28, 29, 31, 32
31	81.3, CH	3.85, m	30, 32	(s) 29, 30, 37, 38, 47; (w) 32, 44	29, 30, 32, 33, 47
32	42.2, CH	1.43, dq (9.4, 6.8, 6.8, 6.8)	31, 33, 47	(s) 30, 33, 34b, 47; (w) 31	30, 31, 33, 34, 47
33	81.3, CH	4.30, d (6.5)	32, 34a	(s) 32, 34a, 34b, 47	31, 34, 35, 36, 47

34a	39.9, CH2	2.05, ddd (11.6, 6.6, 2.7)	33, 34b, 35	(s) 33, 34b, 35, 36	32, 33
34b		1.92, dd (11.6, 1.3)	34a, 35	(s) 32, 33, 34a, 35	32, 33, 35, 36
35	77.7, CH	4.40, m	34a, 34b, 36	(s) 34a, 34b, 36, 37; (w) 38	31, 33, 34, 36
36	82.2, CH	4.59, br m	35, 37, 38	(s) 34a, 35, 37, 38	37, 38, 39
37	135.6, CH	6.24, dd (15.3, 3.9)	36, 38, 39	(s) 31, 35, 36, 39	35, 36, 38, 39
38	127.5, CH	7.03, ddd (15.2, 11.5, 1.5)	36, 37, 39	(s) 31, 36, 41; (w) 35	36, 37, 39, 40
39	138.1, CH	6.46, br dd (10.8, 9.7)	37, 38, 40, 42	(s) 37, 40	37, 38, 41, 43
40	126.6, CH	7.17, m	39	(s) 39; (w) 7	38, 42, 43
41	139.2, CH	7.16, m	42	(s) 38, 42	38, 39, 42, 43
42	119.7, CH	5.62, br d (9.7)	39, 41	(s) 41	39, 40, 43
43	167.8, C				
44	15.7, CH3	0.91, d (6.9)	6	(s) 4, 5, 6, 7; (w) 31	5, 6, 7
45	14.4, CH3	1.67, d (0.9)	7, 9	(s) 6, 9, 10; (w) 5	6, 7, 8, 9, 10, 44
46	10.9, CH3	0.87, d (7.1)	26	(s) 24, 25, 26, 28a, 28b; (w) 30	25, 26, 27
47	15.6, CH3	0.83, d (6.9)	32	(s) 31, 32, 33; (w) 26, 29, 30	31, 32, 33

[a] Acquired in methanol-d_4 at 176.1 MHz and calibrated to solvent signal at 49.2 ppm.
[b] Acquired in methanol-d_4 at 700.4 MHz and calibrated to solvent signal at 3.31 ppm.
[c] Proton showing COSY correlations to indicated proton.
[d] Proton showing ROESY correlations to indicated proton.
[e] Proton showing HMBC correlations to indicated carbon.
[f] Strong ROESY correlations.
[g] Weak ROESY correlations.

Table S2. ^{13}C NMR comparison neosorangicin A (**1**) and sorangicin A (**3**) in methanol-*d*$_4$.

Position	δ ^{13}C [ppm] NeoA (1)	Position	δ ^{13}C [ppm] SorA (3)	Δ (NeoA-SorA)
4	19.3	4	28.2	-8.9
5	71.8	5	38.5	33.3
6	40.3	6	33.0	7.3
7	130.0	7	134.2	-4.1
8	133.4	8	131.2	2.2
9	74.3	9	74.4	-0.1
10	66.9	10	66.9	0.0
11	123.9	11	123.8	0.1
12	137.1	12	136.9	0.2
13	75.3	13	75.3	0.0
14	35.5	14	35.5	0.1
15	128.5	15	128.3	0.1
16	134.0	16	133.6	0.4
17	33.9	17	33.4	0.6
18	34.7	18	34.0	0.8
19	134.6	19	134.4	0.3
20	129.9	20	130.2	-0.3
21	74.5	21	74.4	0.1
22	77.6	22	77.8	-0.1
23	75.1	23	75.1	0.0
24	31.1	24	30.9	0.3
25	71.2	25	71.1	0.1
26	38.3	26	38.5	-0.2
27	74.8	27	74.9	0.0
28	37.2	28	37.1	0.1
29	133.0	29	133.0	0.0
30	133.0	30	132.8	0.2
31	81.3	31	81.2	0.1
32	42.2	32	42.2	0.1
33	81.3	33	81.0	0.2
34	39.9	34	39.9	0.0
35	77.7	35	77.6	0.1
36	82.2	36	82.3	0.0
37	135.6	37	134.9	0.7
38	127.5	38	127.8	-0.3
39	138.1	39	137.6	0.5
40	126.6	40	127.0	-0.4
41	139.2	41	139.1	0.1
42	119.7	42	119.7	0.0
43	167.8	43	167.7	0.2
44	15.7	44	21.7	-6.0
45	14.4	45	14.3	0.1
46	10.9	46	10.9	0.0
47	15.6	47	15.4	0.3

Table S3. ^{1}H NMR comparison neosorangicin A (**1**) and sorangicin A (**3**) in methanol-d_4.

Position	δ ^{1}H [ppm] NeoA (1)	Position	δ ^{1}H [ppm] SorA (2)	Δ (NeoA-SorA)
4a		4a	1.36	
4b	1.01	4b	1.3	-0.29
5a	3.56	5a	1.42	2.14
5b	3.56	5b	1.25	2.31
6	2.53	6	2.43	0.10
7	5.39	7	5.34	0.04
8		8		0.00
9	4.25	9	4.28	-0.03
10	5.32	10	5.35	-0.03
11	6.03	11	6.05	-0.02
12	6.14	12	6.17	-0.03
13	4.41	13	4.43	-0.02
14a	2.39	14a	2.43	-0.05
14b	2.15	14b	2.17	-0.02
15	5.53	15	5.58	-0.05
16	5.53	16	5.58	-0.05
17a	2.15	17a	2.24	-0.09
17b	2.08	17b	2.14	-0.06
18a	2.16	18a	2.24	-0.08
18b	2.08	18b	2.17	-0.09
19	5.72	19	5.79	-0.07
20	5.58	20	5.64	-0.06
21	4.16	21	4.19	-0.03
22	3.47	22	3.52	-0.06
23	3.64	23	3.73	-0.09
24a	1.73	24a	1.76	-0.03
24b	1.64	24b	1.7	-0.06
25	3.85	25	3.87	-0.02
26	1.55	26	1.59	-0.04
27	3.83	27	3.89	-0.06
28a	2.25	28a	2.32	-0.07
28b	2.16	28b	2.17	-0.02
29	5.45	29	5.54	-0.09
30	5.37	30	5.42	-0.05
31	3.85	31	3.87	-0.02
32	1.43	32	1.46	-0.03
33	4.30	33	4.32	-0.02
34a	2.05	34a	2.09	-0.04
34b	1.92	34b	1.97	-0.05
35	4.40	35	4.45	-0.05
36	4.59	36	4.61	-0.02
37	6.24	37	6.26	-0.02
38	7.03	38	7.03	0.00
39	6.46	39	6.48	-0.02
40	7.17	40	7.24	-0.07
41	7.16	41	7.19	-0.03
42	5.62	42	5.66	-0.04
43		43		0.00
44	0.91	44	0.93	-0.02
45	1.67	45	1.68	-0.01
46	0.87	46	0.93	-0.06
47	0.83	47	0.86	-0.03

Table S4. NMR spectroscopic data of neosorangioside A (**3**) acquired at 700/175 MHz in methanol-*d₄*.

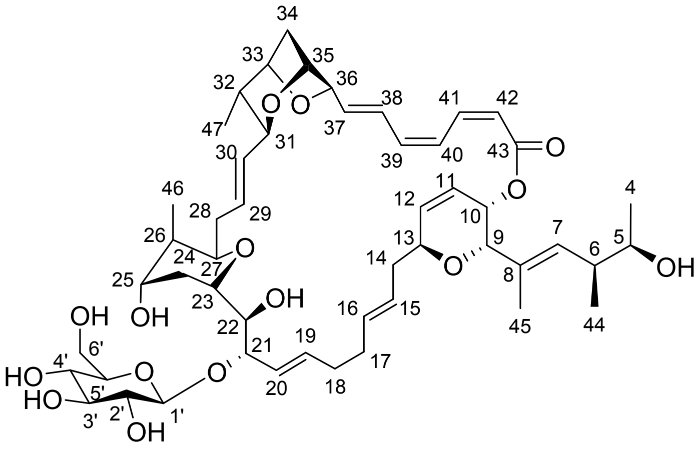

Position	δ_C[a] [ppm], type	δ_H[b] [ppm], mult	COSY[c]	ROESY[d]	HMBC[e]
4	19.2, CH3	1.00, d (6.2)	5	5, 6, 7, 44	5, 6
5	71.7, CH	3.55, qd (6.4, 6.4, 6.4, 4.4)	4, 6	4, 6, 7, 44	4, 6, 7, 44
6	40.2, CH	2.53, dqd (10.0, 6.7, 6.7, 6.7, 4.3)	44, 5, 7, 44	4, 5, 44, 45	4, 5, 7, 8, 44
7	130.1, CH	5.36, m	6, 45	4, 5, 9, 44	5, 6, 8, 9, 44, 45
8	133.3, C				
9	74.3, CH	4.23, s	10, 45	7, 10, 14a, 45	7, 8, 10, 13, 45
10	66.9, CH	5.35, dd (5.8, 1.7)	9, 11	9, 11, 45	11, 12, 14, 43
11	124.0, CH	6.03, ddd (9.9, 5.8, 2.2)	10, 12, 13	10	10, 12, 13
12	137.2, CH	6.14, dd (10.1, 3.0)	11, 13	13, 14b	10, 11, 13
13	75.2, CH	4.42, dq (11.2, 3.1, 3.1, 3.1)	11, 12, 14a, 14b	12, 14b, 15, 16	9, 11, 12, 14, 15
14a	35.5, CH2	2.38, ddd (14.0, 10.9, 8.0)	13, 14b, 15	9, 14a, 15, 16	12, 13, 15, 16
14b		2.14, m	13, 14a, 15	12, 13, 14a, 15, 16	
15	128.5, CH	5.54, ddd (15.3, 8.4, 5.2)	14a, 14b, 16	13, 14a, 14b, 17	13, 14, 16, 17
16	134.1, CH	5.51, ddd (15.3, 7.3, 4.3)	15, 17a, 17b	13, 14a, 14b	14, 17
17a	34.1, CH2	2.15, m	16, 17b, 18b	17b, 20	15, 16, 18, 19, 20
17b		2.06, m	16, 17a, 18a	15, 17a, 19	15, 18, 19
18a	35.1, CH2	2.15, m	17b, 18b, 19, 20	18b, 20	15, 16, 17, 19, 20
18b		2.05, m	18a, 19, 20	18a, 19	16, 17, 19, 20
19	136.4, CH	5.78, ddd (15.3, 7.7, 5.6)	18a, 18b, 20	17b, 18b, 21	17, 18, 21
20	127.1, CH	5.61, br d (9.5)	18a, 18b, 19, 21	17a, 18a, 21, 23	18, 21, 22
21	83.3, CH	4.34, dd (8.4, 3.0)	20, 22	19, 20, 22, 23	19, 20, 1'
22	75.6, CH	3.68, m	21, 23	1', 21, 24	20, 21, 23, 24
23	74.3, CH	3.60, ddd (11.5, 8.5, 2.2)	22, 24a, 24b	20, 21, 24a, 27	21, 22, 25
24a	31.6, CH2	1.79, br d (14.0)	23, 24b, 25	23, 24b, 25	25, 26
24b		1.60, ddd (14.3, 11.7, 2.9)	23, 24a, 25	22, 24a, 25, 46	23
25	71.2, CH	3.85, m	24a, 24b, 26	24a, 24b, 26, 46, 47	23, 46
26	38.0, CH	1.54, m	25, 27, 46	25, 27, 30, 46, 47	24, 25, 46
27	74.6, CH	3.81, m	26, 28a, 28b	23, 26, 28a, 30	28, 46
28a	37.2, CH2	2.26, ddd (13.6, 5.0, 2.2)	27, 28b, 30	27, 28b, 29	26, 27, 29
28b		2.18, m	27, 28a, 29, 30	28a, 29, 46	27, 29
29	132.6, CH	5.38, m	28b, 31	28a, 28b, 31, 32, 47	28, 31
30	133.1, CH	5.38, m	28a, 28b, 31	26, 27, 31, 46	
31	81.3, CH	3.85, m	29, 30, 32	29, 30, 37, 46, 47	29, 32. 47
32	42.2, CH	1.42, dq (9.6, 6.8, 6.8, 6.8)	31, 33, 47	29, 33, 34b, 47	30, 31, 33, 34, 47

33	81.3, CH	4.31, d (6.7)	32, 34a, 34b	32, 34a, 34b, 47	31, 34, 35, 36, 47
34a	39.8, CH2	2.04, m	33, 34b, 35	33, 34b, 35, 36	31, 32, 33
34b		1.93, dd (11.5, 1.4)	33, 34a, 35	32, 33, 34a, 35	32, 35, 36
35	77.7, CH	4.41, br m	34a, 34b, 36	34a, 34b, 36, 37	31
36	82.1, CH	4.60, br q (2.6, 2.6, 2.6)	35, 37, 38	34a, 35, 37, 38	37, 38
37	135.7, CH	6.25, dd (15.3, 3.7)	36, 38, 39	31, 35, 36, 39	35, 36, 38, 39
38	127.5, CH	7.04, ddd (15.1, 11.5, 1.6)	36, 37, 39	36, 41	36, 39, 40
39	138.1, CH	6.46, m	37, 38, 40, 42	37, 40	37, 38, 41, 43
40	126.6, CH	7.16, m	39	39	38, 42, 43
41	139.2, CH	7.16, m	42	38, 42	39, 43
42	119.7, CH	5.61, m	39, 41	41	40, 43
43	167.8, C				
44	15.6, CH3	0.90, d (6.9)	6	4, 5, 6, 7, 45	5, 6, 7
45	14.4, CH3	1.66, d (0.9)	7, 9	6, 9, 10, 44	6, 7, 8, 9, 10, 44
46	10.8, CH3	0.87, d (7.1)	26	24b, 25, 26, 28, 30, 31	25, 26, 27
47	15.7, CH3	0.83, d (6.7)	32	25, 26, 29, 31, 32, 33	31, 32
1'	102.8, CH	4.36, d (7.7)	2'	22, 3', 5'	21, 5'
2'	75.4, CH	3.21, dd (9.0, 7.7)	1', 3'	3'	1', 3', 4'
3'	78.2, CH	3.35, t (9.0, 9.0)	2', 4'	1', 2', 5'	1', 2', 4'
4'	71.6, CH	3.32, d (9.3)	3', 5'		3', 6'
5'	78.0, CH	3.22, m	4', 6'a, 6'b	1', 3', 6'a, 6'b	1', 4', 6'
6'a	62.8, CH2	3.82, m	5', 6'b	5', 6'b	4', 5'
6'b		3.68, m	5', 6'a	5', 6'a	4', 5'

[a] Acquired in methanol-d_4 at 176.1 MHz and calibrated to solvent signal at 49.2 ppm.
[b] Acquired in methanol-d_4 at 700.4 MHz and calibrated to solvent signal at 3.31 ppm.
[c] Proton showing COSY correlations to indicated proton.
[d] Proton showing ROESY correlations to indicated proton.
[e] Proton showing HMBC correlations to indicated carbon.

Table S5. ^{13}C NMR comparison neosorangioside A (**2**) and sorangioside A (**4**) in methanol-*d*$_4$.

Position	δ ^{13}C [ppm] Neosorangiosid A (2)	Position	δ ^{13}C [ppm] Sorangiosid A (4)	Δ (2-4)
4	19.2	4	28.2	-9.0
5	71.7	5	38.5	33.2
6	40.2	6	32.9	7.3
7	130.1	7	134.3	-4.2
8	133.3	8	131.3	2.0
9	74.3	9	74.4	-0.1
10	66.9	10	66.9	0.0
11	124.0	11	123.9	0.1
12	137.2	12	136.9	0.3
13	75.2	13	76.3	-1.1
14	35.5	14	35.3	0.2
15	128.5	15	129.0	-0.5
16	134.1	16	132.9	1.2
17	34.1	17	33.6	0.5
18	35.1	18	33.4	1.7
19	136.4	19	136.4	0.0
20	127.1	20	127.2	-0.1
21	83.3	21	83.2	0.0
22	75.6	22	76.0	-0.4
23	74.3	23	74.0	0.3
24	31.6	24	31.4	0.2
25	71.2	25	71.0	0.2
26	38.0	26	38.0	0.0
27	74.6	27	74.4	0.2
28	37.2	28	37.1	0.0
29	132.6	29	132.4	0.2
30	133.1	30	133.1	0.0
31	81.3	31	81.2	0.1
32	42.2	32	42.1	0.1
33	81.3	33	81.1	0.2
34	39.8	34	39.8	0.0
35	77.7	35	77.5	0.2
36	82.1	36	82.0	0.1
37	135.7	37	134.9	0.8
38	127.5	38	127.8	-0.4
39	138.1	39	137.6	0.6
40	126.6	40	127.0	-0.4
41	139.2	41	139.1	0.1
42	119.7	42	119.7	0.0
43	167.8	43	167.7	0.2
44	15.6	44	21.7	-6.1
45	14.4	45	14.3	0.1
46	10.8	46	10.6	0.2
47	15.7	47	15.4	0.3
1'	102.8	1'	102.8	0.0
2'	75.4	2'	75.2	0.2
3'	78.2	3'	78.1	0.1
4'	71.6	4'	71.6	0.0
5'	78.0	5'	77.8	0.2
6'	62.8	6'	62.8	0.0

Table S6. Blastp results of the CDS regions in the *nsr* BGC.

CDS Name	Length [AA]	Closest homologue [Organism of origin]	Identity [%] and query coverage [%]	Accession Nr.
**NsrJ**	469	SorJ [Sorangium cellulosum]	90.3; 100	ADN68485.1
**NsrK**	408	SorK [Sorangium cellulosum]	92.7; 100	ADN68486.1
**NsrL**	788	SorL [Sorangium cellulosum]	91.4; 88	ADN68487.1
**NsrM**	1105	SorM [Sorangium cellulosum]	88.8; 100	ADN68497.1
**NsrN**	469	SorN [Sorangium cellulosum]	94.7; 100	ADN68488.1
**NsrO**	617	SorO [Sorangium cellulosum]	90.1; 100	ADN68489.1
**Nsr1**	59	hypothetical protein [Micromonosporaceae bacterium]	61.3; 53	HKE65492.1
**Nsr2**	88	acyl carrier protein [Steroidobacteraceae bacterium]	62.7; 94	MGH8260247.1
**NsrQ**	661	SorQ [Sorangium cellulosum]	93.3; 100	ADN68491.1
**NsrA**	8254	too big to be analyzed		
**NsrB**	4970	SorB [Sorangium cellulosum]	91.9; 100	ADN68477.1
**NsrC**	2076	SorC [Sorangium cellulosum]	90.7; 100	ADN68478.1
**NsrD**	3192	SorD [Sorangium cellulosum]	92.1; 100	ADN68479.1
**NsrE**	5314	SorE [Sorangium cellulosum]	91.7; 100	ADN68480.1
**NsrF**	424	SorF [Sorangium cellulosum]	93.2; 100	ADN68481.1
**NsrGH**	6699	SDR family NAD(P)-dependent oxidoreductase [Pendulispora brunnea]	69.0; 95	WP_394849115.1
		SorH [Sorangium cellulosum]	92.6; 90	ADN68483.1
**NsrI**	2610	SorI [Sorangium cellulosum]	91.5; 100	ADN68484.1
**NsrR**	383	SorR [Sorangium cellulosum]	93.2; 100	ADN68492.1
**NsrS**	404	SorS [Sorangium cellulosum]	92.3; 100	ADN68493.1
**NsrT**	584	SorT [Sorangium cellulosum]	86.1; 100	ADN68494.1
**NsrU**	461	MATE family efflux transporter [Sorangium sp.]	96.5; 100	HTN89125.1
		SorU [Sorangium cellulosum]	83.2; 99	ADN68498.1

Table S7. Analysis of domains in the *nsr* BGC in *S. cellulosum* Soce417. Substrate specificities of acyl-transferase (AT) domains are based on antiSMASH[1] predictions, comparison to the AT domains in the *sor* BGC[2] (Figure S3) and fingerprint analysis of acyl hydrolase domains[3]. Functionality of ACP domains was determined based on fingerprint analysis.[4] Analysis of the ketosynthase (KS) domains was done based on phylogeny with the transATor tool[5] and clades with the highest score were listed. The stereochemistry predictions for the dehydratase (DH) domains are based on the orientation of the respective hydroxy-group[4]. The listed stereochemistries for the ketoreductase (KR) domains are based on antiSMASH predictions. Activities of all domains were evaluated based on reported fingerprints of active sites.[4,6] Additional modules compared to the biosynthetic machinery in the *sor* BGC are marked bold.

Gene	Module	Domain	Characterization/comment
NsrO	Trans-AT	AT_a	Malonyl-CoA; Hydroxy-malonlyl-CoA, Acyl Hydrolase domain
		AT_b	Malonyl-CoA
NsrA	1	KS	Clade 8: unusual starter: AMT/succinate
		DH	Stereochemistry unclear
		ACP	
	2	KS	Clade 25: completely reduced
		**DH**	Stereochemistry unclear
		ACP_a	
		ACP_b	
	3	KS	Clade 74: α-Me reduced/keto/D-OH
		DH	*E*-configured double bond
		KR	D-configured hydroxy-group
		C-MT	
		**ACP_a**	
		ACP_b	
	4	KS	Clade 113: double bonds (*E*-configured; (some with α-Me)
		KR	L-configured hydroxy-group
		ACP	
	5	KS	Clade 66: β-L-OH
		ACP_a	
		**ACP_b**	
	6	KS	Clade 73: exomethylene
		KR	L-configured hydroxy-group
		ACP	
	7	KS[a]	Clade 31: non-elongating (bimodule β-D-OH)
NsrB		DH	
		ACP	
		DH[b]	Inactive
	8	KS[a]	Clade 76: non-elongating (double bonds)
		PS	
		ACP	
	9	KS	Clade 26: pyran/furan rings
		DH	Stereochemistry unclear
		ACP	
	10	KS	Clade 82: double bonds (mostly *E*-configured)
		DH	*E*-configured double bond
		KR	D-configured hydroxy-group
		ACP	
	11	KS	Clade 25: completely reduced
NsrC		DH	*E*-configured double bond
		**KR**	D-configured hydroxy-group
		ACP	
	12	KS	Clade 113: double bonds (*E*-configured; (some with α-Me)
		KR	L-configured hydroxy-group
NsrD		C-MT	
		ACP_a	

		ACP_b	
	13	KS	Clade 68 α-L-OH/Me-β-D-OH
		KR	L-configured hydroxy-group
		ACP_a	
		**ACP_b**	
	14	KS	Clade 66: β-L-OH
		KR	L-configured hydroxy-group
		C-MT	
		ACP_a	
		**ACP_b**	
	15	KS	Clade 74: α-Me reduced/keto/D-OH
		DH	*E*-configured double bond
		PS	
		KR	D-configured hydroxy-group
NsrE		ACP	
	16	KS	Clade 26: pyran/furan rings
		DH	*E*-configured double bond
		KR	D-configured hydroxy-group
		ACP	
	17	KS	Clade 82: double bonds (mostly *E*-configured)
		DH	*Z*-configured double bond
		KR	L-configured hydroxy-group
		C-MT	
		ACP	
	18	KS	Clade_68 α-L-OH/Me-β-D-OH
		**O-MT**	
		ACP	
	19	KS	Clade 103: β-OMe
		DH	*E*-configured double bond
		PS	
NsrGH		KR	D-configured hydroxy-group
		ACP	
	20	KS	Clade 26: pyran/furan rings
		DH	*E*-configured double bond
		KR	D-configured hydroxy-group
		ACP	
	21	KS	Clade 82: double bonds (mostly *E*-configured)
		KR	L-configured hydroxy-group
		ACP	
	22	KS[a]	Clade 31: non-elongating (bimodule β-D-OH)
		DH	*Z*-configured double bond
		ACP	
	23	KS	Clade 82: double bonds (mostly *E*-configured)
NsrI		DH	*Z*-configured double bond
		KR	L-configured hydroxy-group
		ACP	
		KS[a]	Clade 76: non-elongating (double bonds)
		DH[b]	Inactive

[a] KS domains have an incomplete CHH catalytic triad and are therefore inactive.
[b] The DH domains are inactive, indicated by the incomplete **H**xxxGxxxxP motif and the missing aspartic acid in an HPALL**D** motif.

Supplementary Figures

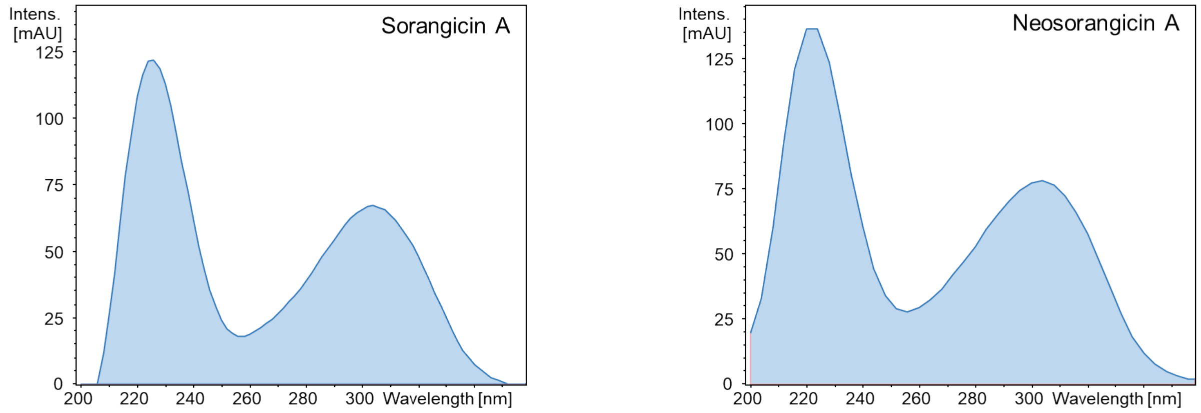

Figure S1. UV-spectra of sorangicin A and neosorangicin A in methanolic crude extracts of standard cultivations of *S. cellulosum* strains.

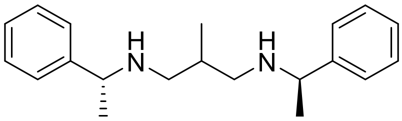

Figure S2. Chemical structure of Bis-1,3-methylbenzylamine-2-methylpropane (BMBA-*p*-Me).

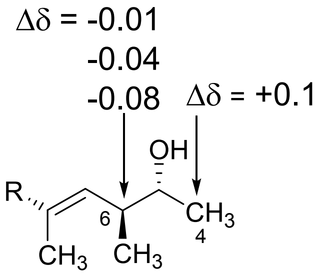

Figure S3. ^{13}C Chemical shift differences of the carbon atoms next to the new stereocenter of neosorangicin A (**1**) in the chiral NMR solvents BMBA-*p*-Me: $\Delta\delta = \delta_{(R,R)\text{-BMBA}} - \delta_{(S,S)\text{-BMBA}}$ in ppm.

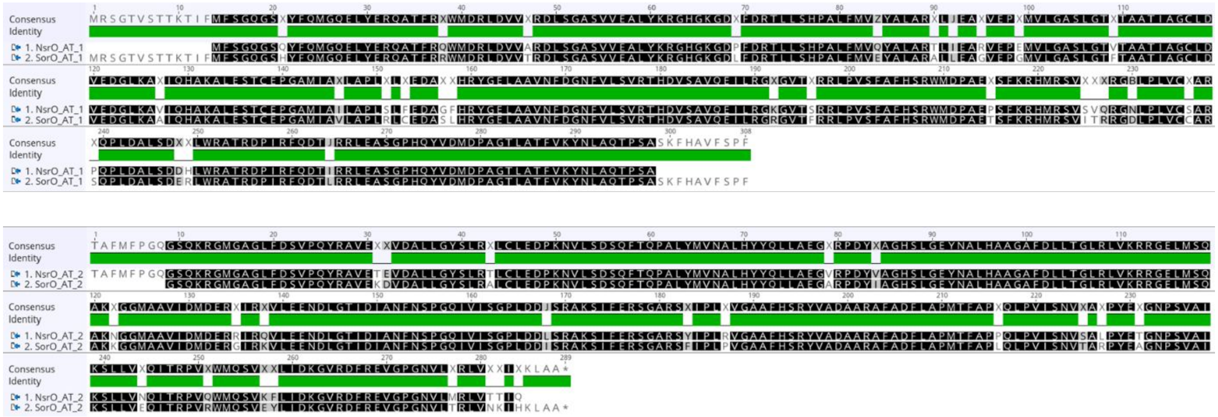

Figure S4. Alignment of the AT domains in NsrO in the *nsr* BGC with the ones in SroO in the *sor* BGC. The respective AT domains display a high similarity with 90.2% identity for AT 1 and 91.7% identity for AT 2.

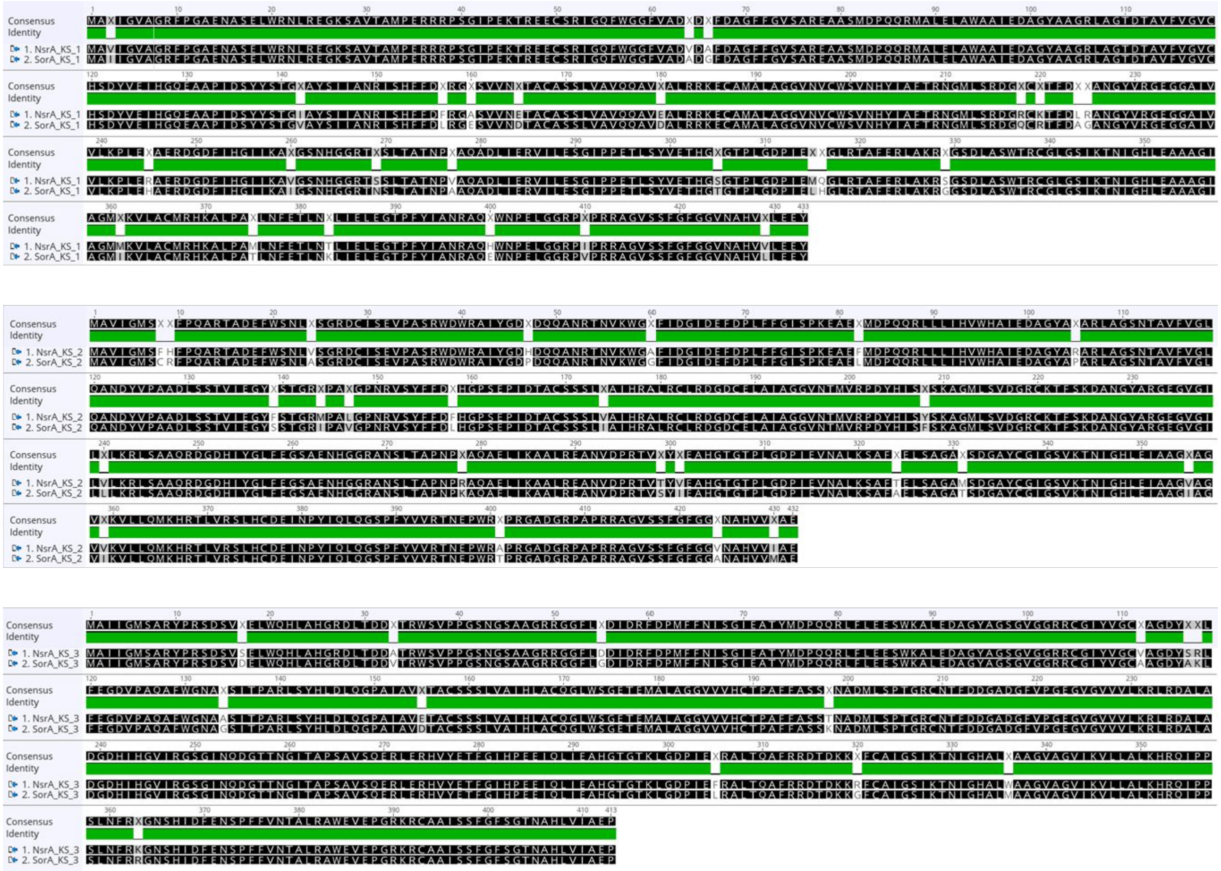

Figure S5. Alignment of the KS domains in modules 1-3 in NsrA and SroA. The respective KS domains show a high similarity with 94.0% identity for KS 1, 94.4% for KS 2 and 96.9% for KS 3.

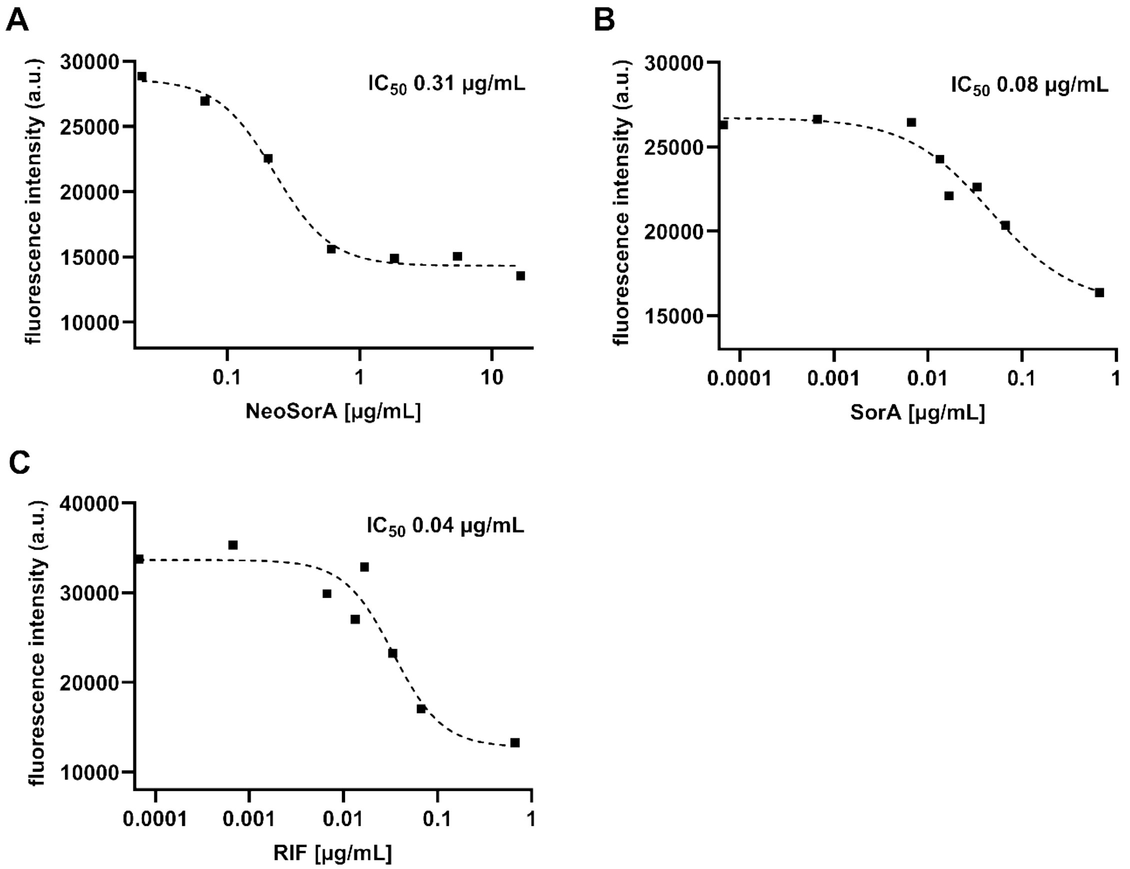

Figure S6. *S. aureus* RNA polymerase inhibition assays. Data were plotted using GraphPad Prism (version 10.2.3) and IC_{50} values were determined by sigmoidal curve fitting (**A** neosorangicin A, **B** sorangicin A, **C** rifampicin). NeoSorA: neosorangicin A; RIF: rifampicin; SorA: sorangicin A.

A

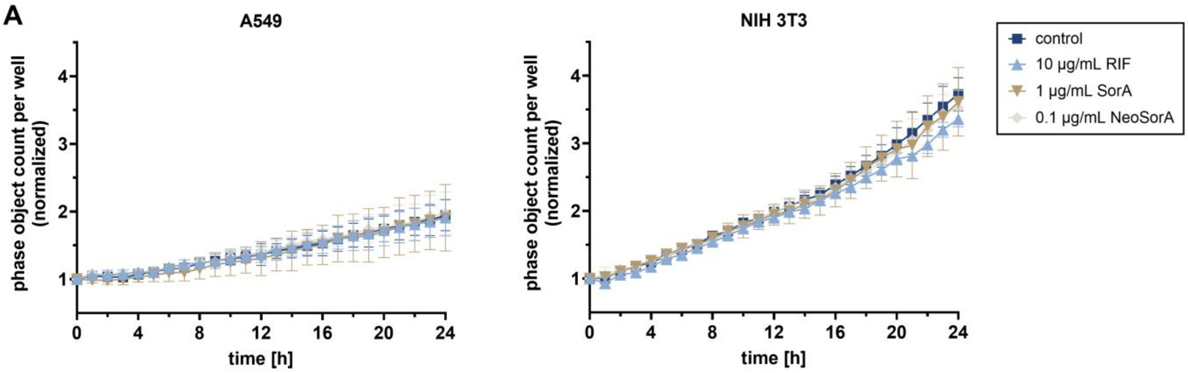

B

	IC_{50} [µg/mL]		
	0.1% MeOH	SorA	NeoSorA
**A549**	-	22.5	15
**NIH 3T3**	-	28	19

Figure S7. A Growth curves of A549 and NIH 3T3 cells treated with either 0.1% (*v/v*) methanol, 1 µg/mL sorangicin A (SorA), 0.1 µg/mL neosorangicin A (NeoSorA), or 10 µg/mL rifampicin (RIF). Cells were seeded at $3x10^3$ cells per well in 96-well plates and monitored over 24 hours by live-cell imaging. Object counts were quantified using the Adherent Cell-by-Cell Analysis module and normalized to the initial cell number. Data represent quadruplicate wells. **B** IC_{50} values of the indicated compounds for A549 and NIH 3T3 cells as determined by MTT assay.

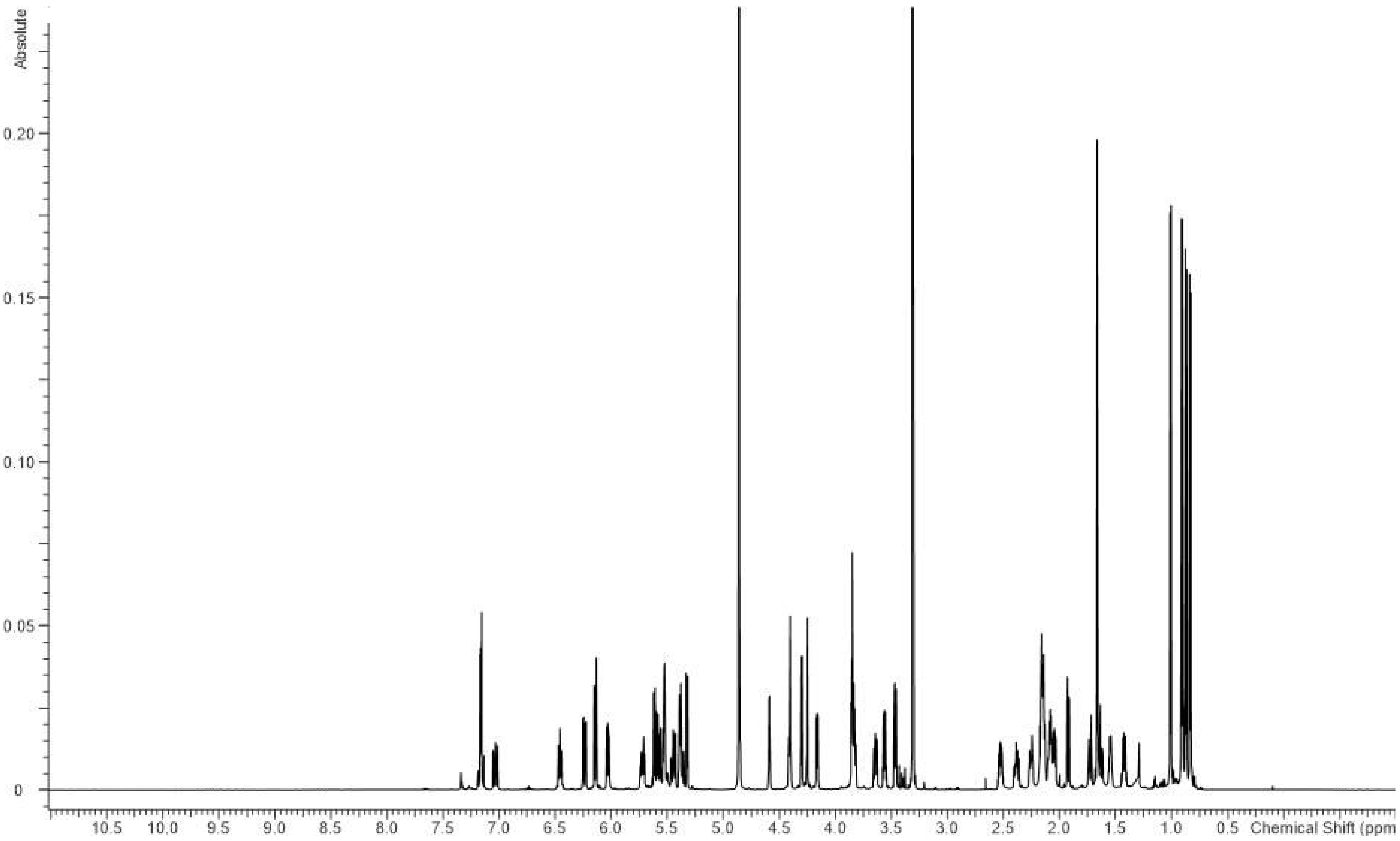

Figure S8. ^{1}H-NMR spectrum of neosorangicin A (**1**) in methanol-d_4 (700.4 MHz).

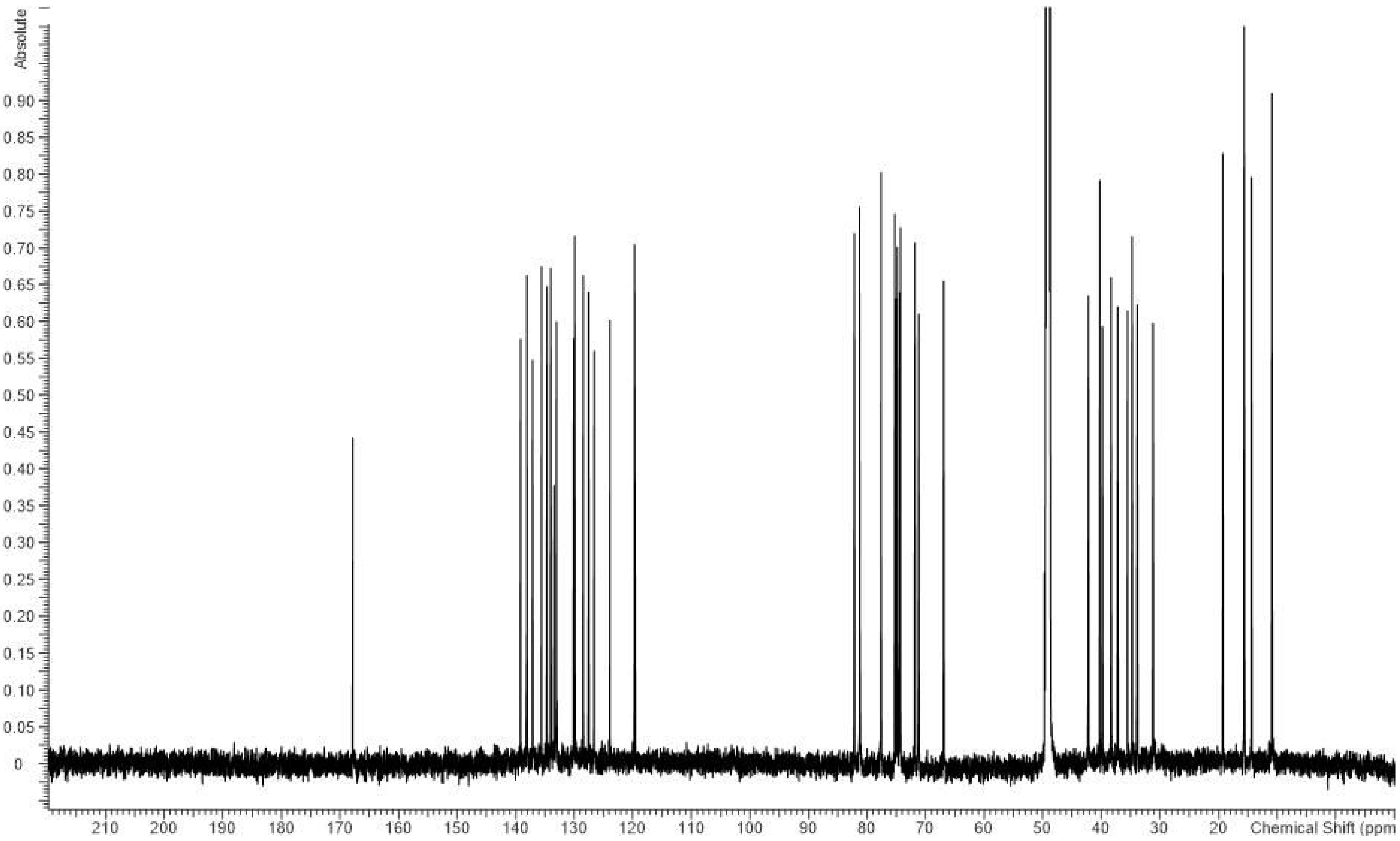

Figure S9. ^{13}C-NMR spectrum of neosorangicin A (**1**) in methanol-d_4 (176.1 MHz).

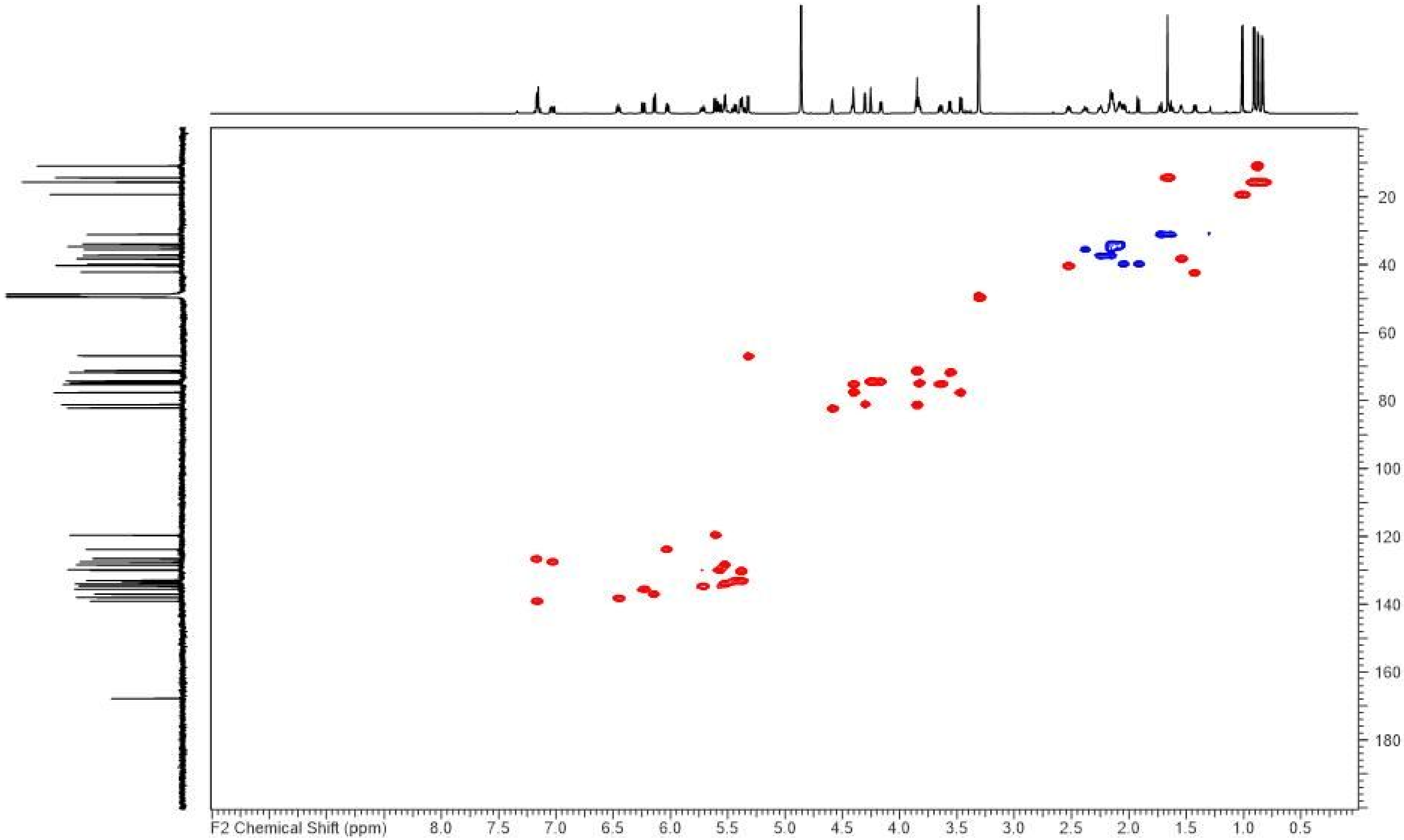

Figure S10. HSQC NMR spectrum of neosorangicin A (**1**) in methanol-*d*$_4$ (176.1/700.4 MHz).

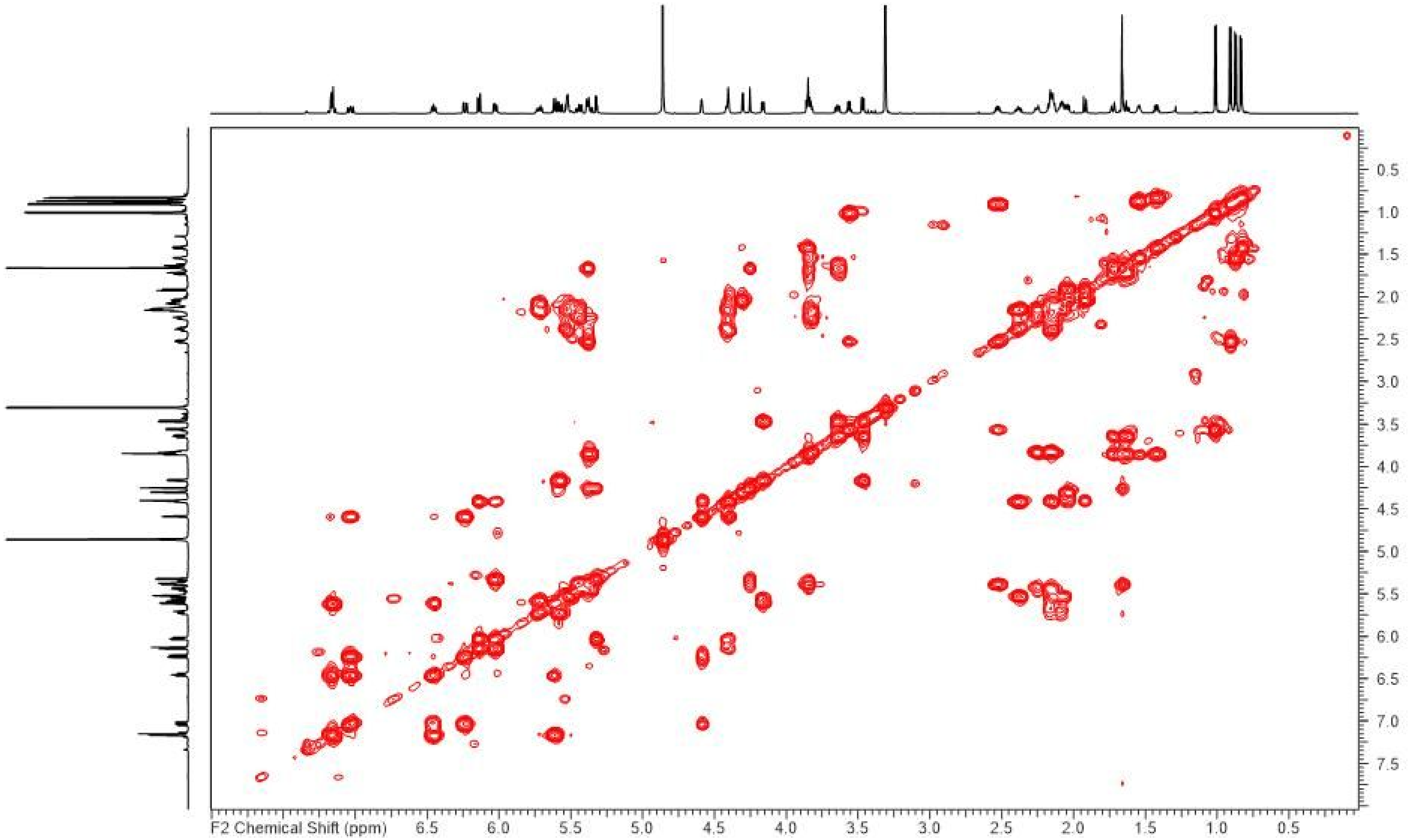

Figure S11. COSY NMR spectrum of neosorangicin A (**1**) in methanol-d_4 (700.4 MHz).

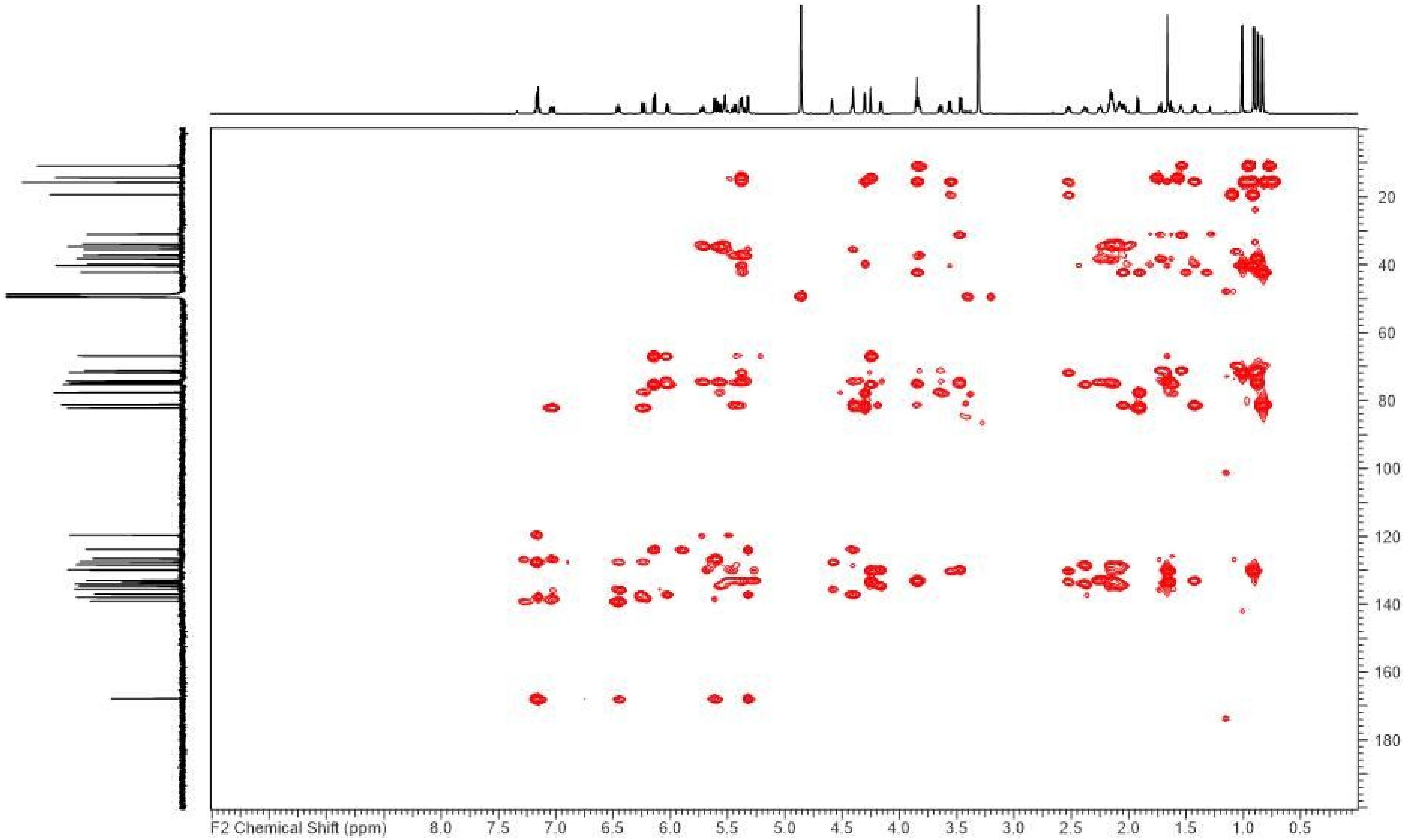

Figure S12. HMBC NMR spectrum of neosorangicin A (**1**) in methanol-d_4 (176.1/700.4 MHz).

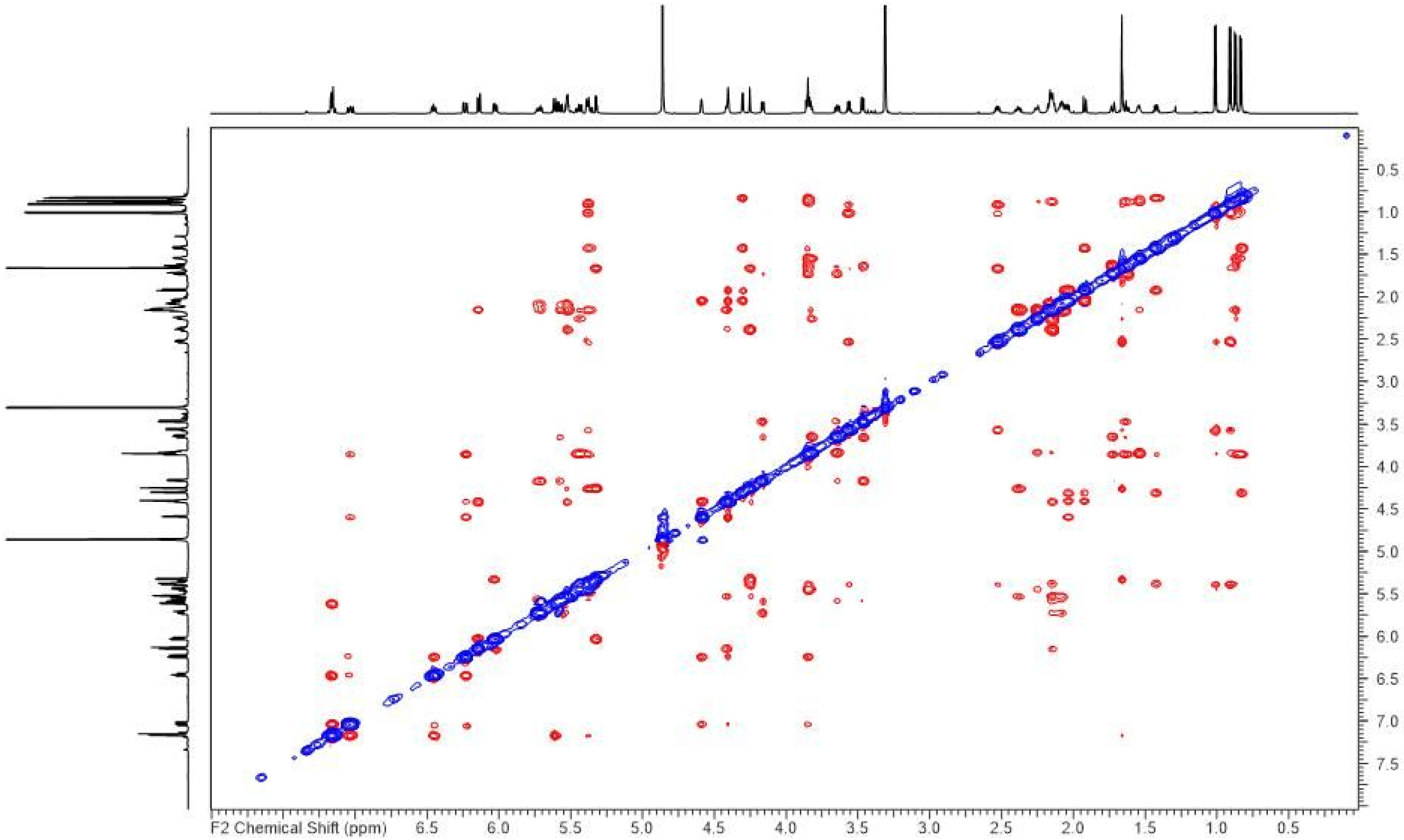

Figure S13. ROESY NMR spectrum of neosorangicin A (**1**) in methanol-d_4 (700.4 MHz).

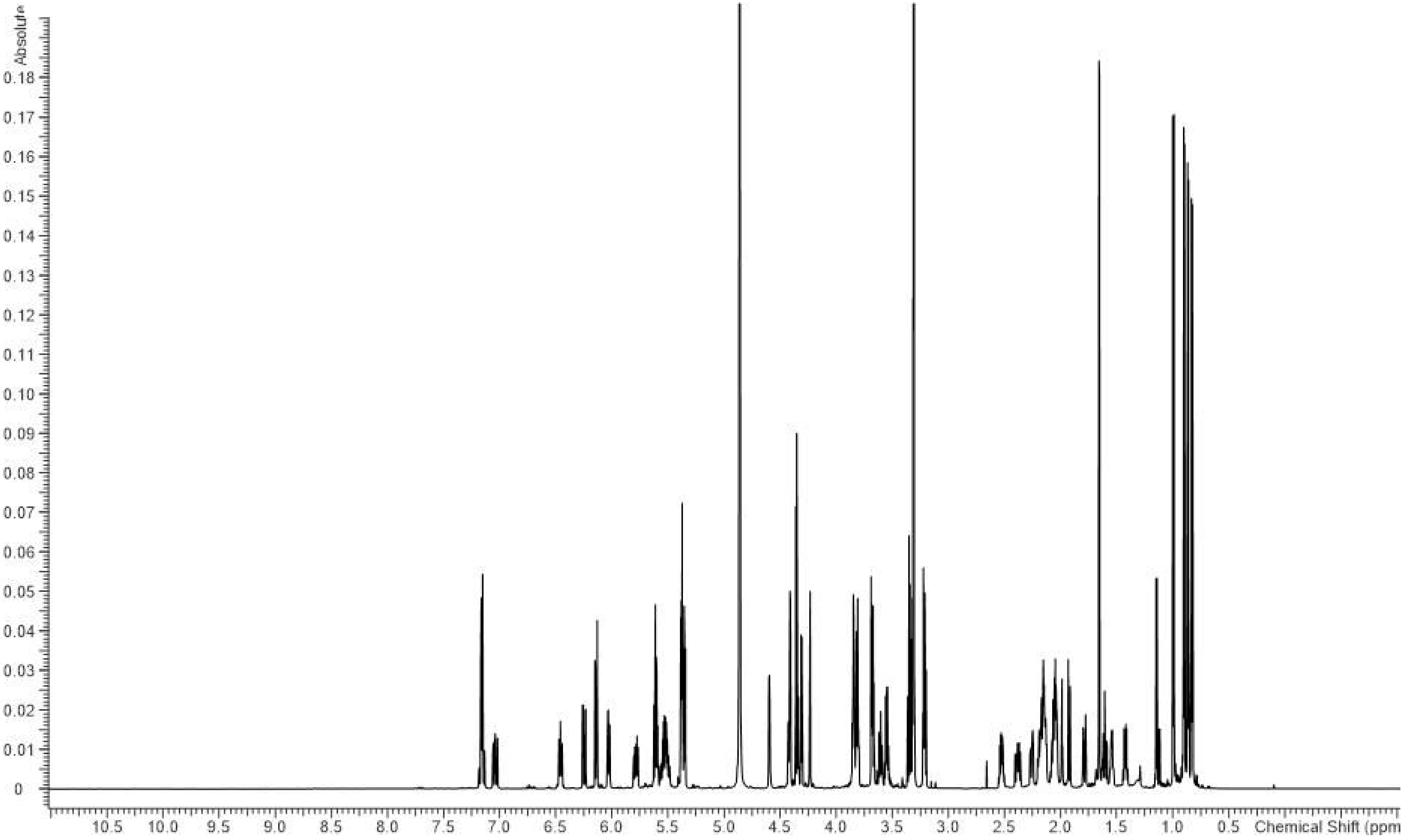

Figure S14. ^{1}H-NMR spectrum of neosorangioside A (**2**) in methanol-d_4 (700.4 MHz).

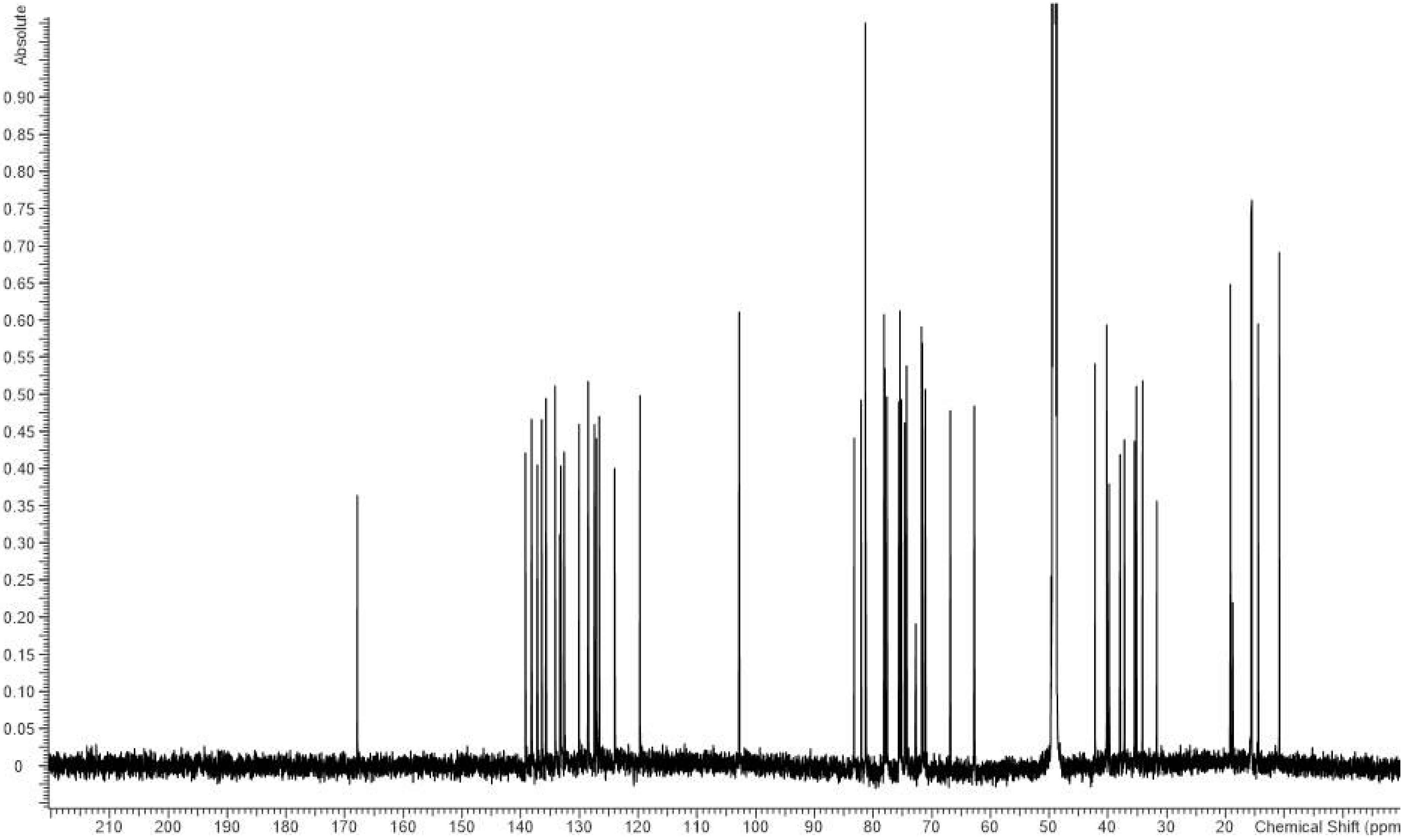

Figure S15. ^{13}C-NMR spectrum of neosorangioside A (**2**) in methanol-d_4 (176.1 MHz).

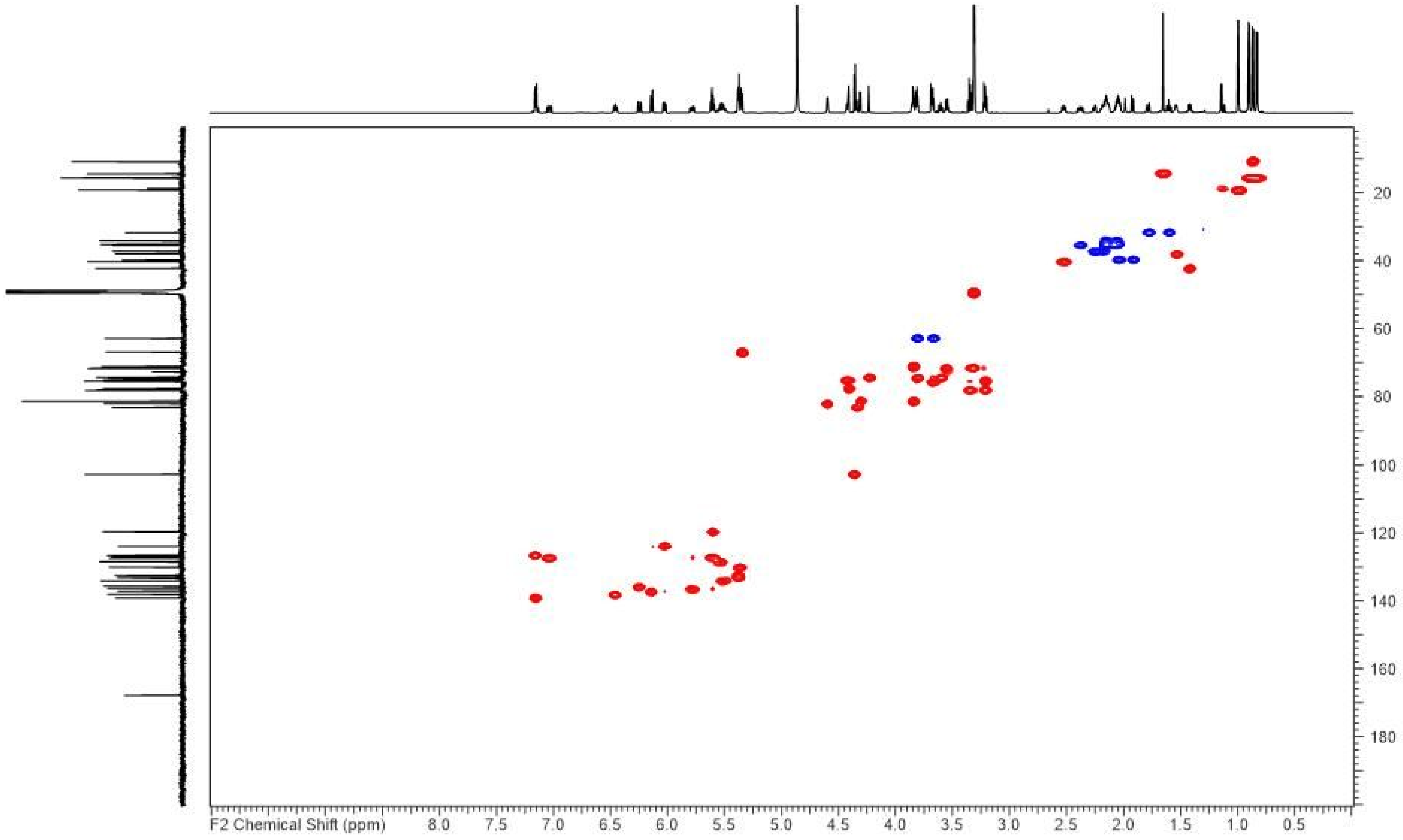

Figure S16. HSQC NMR spectrum of neosorangioside A (**1**) in methanol-d_4 (176.1/700.4 MHz).

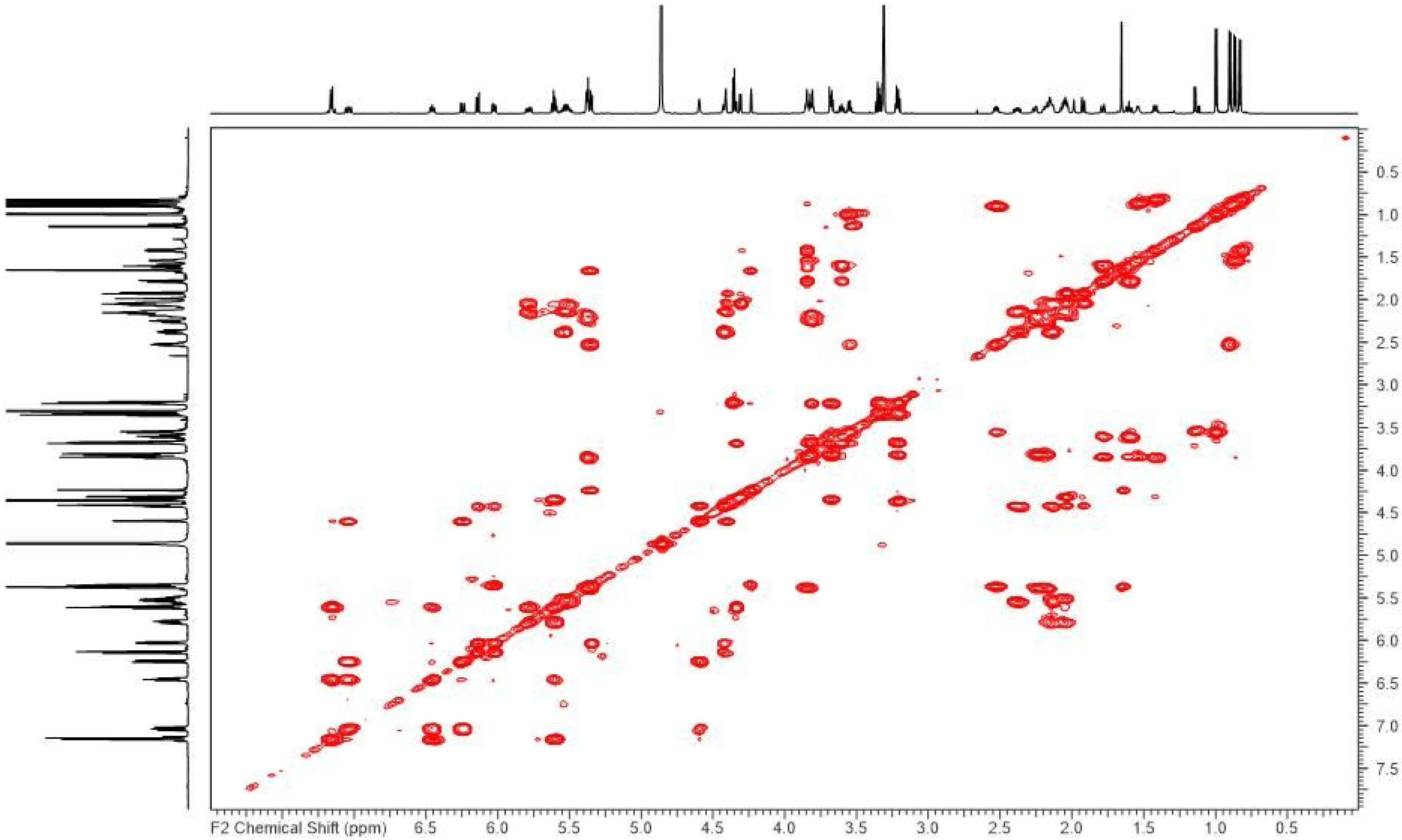

Figure S17. COSY NMR spectrum of neosorangioside A (**1**) in methanol-d_4 (700.4 MHz).

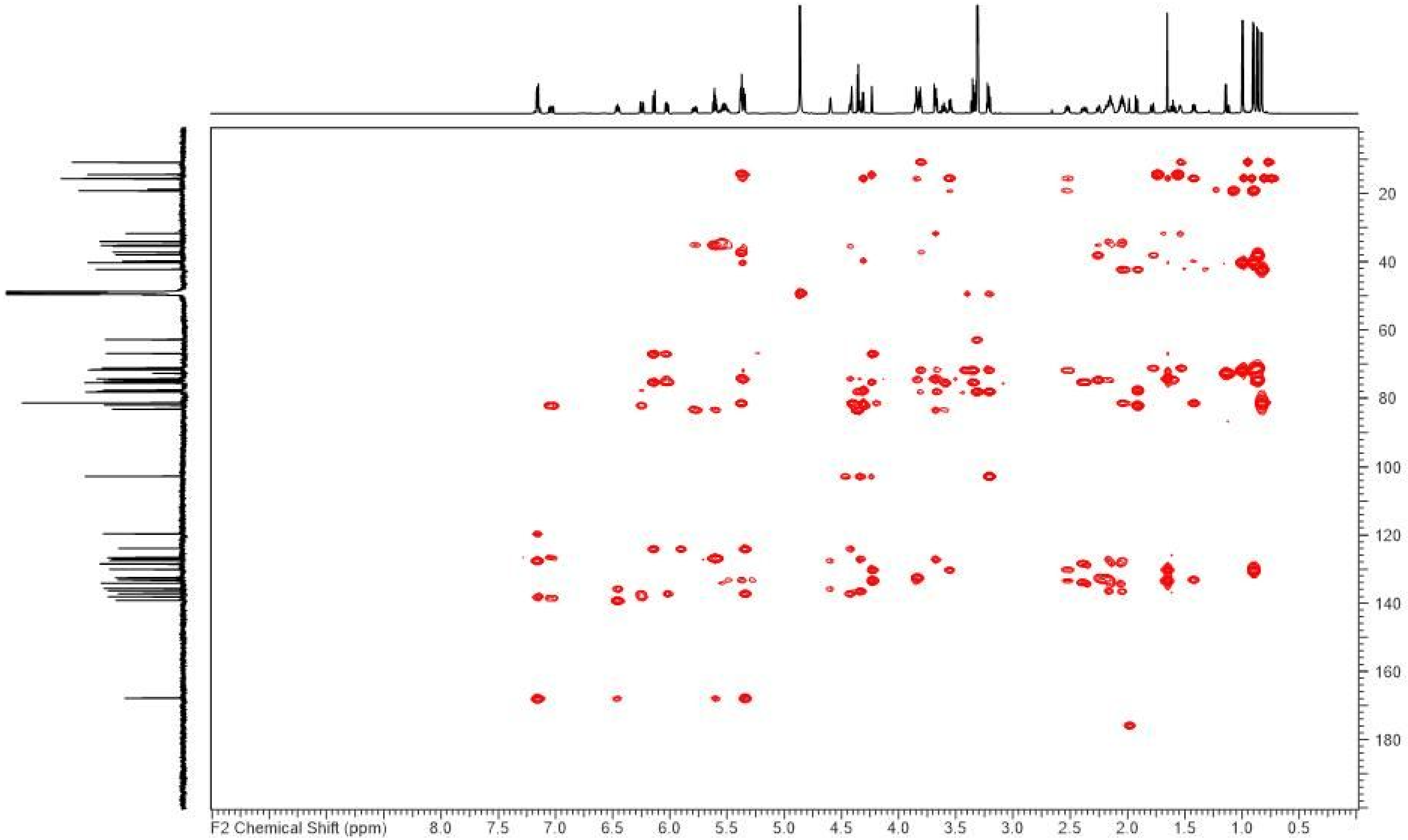

Figure S18. HMBC NMR spectrum of neosorangioside A (**1**) in methanol-d_4 (176.1/700.4 MHz).

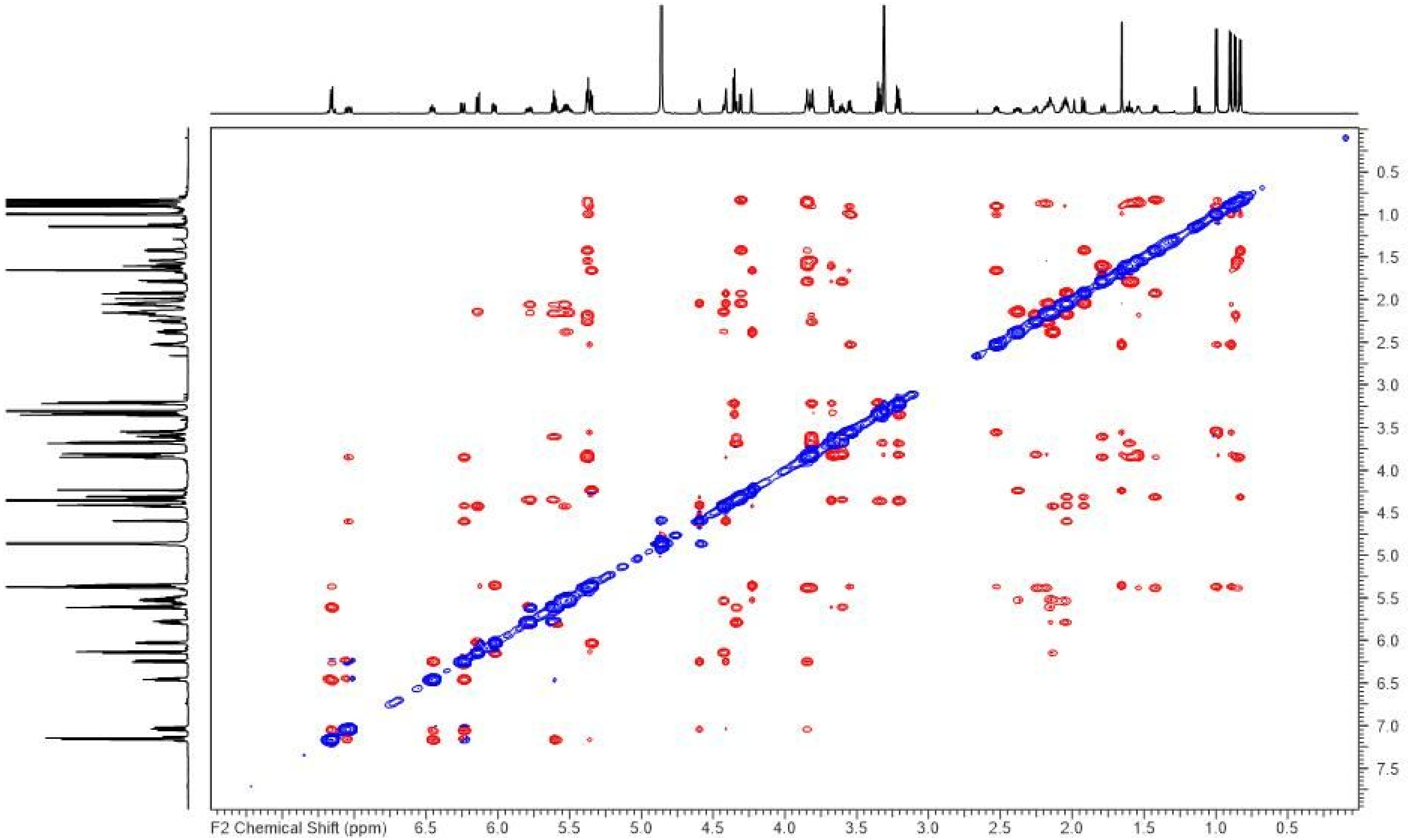

Figure S19. ROESY NMR spectrum of neosorangioside A (**1**) in methanol-d_4 (700.4 MHz).

References

(1) Blin, K.; Shaw, S.; Vader, L.; Szenei, J.; Reitz, Z. L.; Augustijn, H. E.; Cediel-Becerra, J. D. D.; de Crécy-Lagard, V.; Koetsier, R. A.; Williams, S. E.; Cruz-Morales, P.; Wongwas, S.; Segurado Luchsinger, A. E.; Biermann, F.; Korenskaia, A.; Zdouc, M. M.; Meijer, D.; Terlouw, B. R.; van der Hooft, J. J. J.; Ziemert, N.; Helfrich, E. J. N.; Masschelein, J.; Corre, C.; Chevrette, M. G.; van Wezel, G. P.; Medema, M. H.; Weber, T. AntiSMASH 8.0: Extended Gene Cluster Detection Capabilities and Analyses of Chemistry, Enzymology, and Regulation. *Nucleic Acids Research* **2025**, *53* (W1), W32–W38. https://doi.org/10.1093/nar/gkaf334.

(2) Irschik, H.; Kopp, M.; Weissman, K. J.; Buntin, K.; Piel, J.; Müller, R. Analysis of the Sorangicin Gene Cluster Reinforces the Utility of a Combined Phylogenetic/Retrobiosynthetic Analysis for Deciphering Natural Product Assembly by *Trans* -AT PKS. *ChemBioChem* **2010**, *11* (13), 1840–1849. https://doi.org/10.1002/cbic.201000313.

(3) Fage, C. D.; Passmore, M.; Tatman, B. P.; Smith, H. G.; Jian, X.; Dissanayake, U. C.; Foran, M. E.; Cisneros, G. A.; Challis, G. L.; Lewandowski, J. R.; Jenner, M. Molecular Basis for Short-Chain Thioester Hydrolysis by Acyl Hydrolases in *Trans* -Acyltransferase Polyketide Synthases. *JACS Au* **2025**, *5* (1), 144–157. https://doi.org/10.1021/jacsau.4c00837.

(4) Keatinge-Clay, A. T. The Structures of Type I Polyketide Synthases. *Nat. Prod. Rep.* **2012**, *29* (10), 1050. https://doi.org/10.1039/c2np20019h.

(5) Helfrich, E. J. N.; Ueoka, R.; Dolev, A.; Rust, M.; Meoded, R. A.; Bhushan, A.; Califano, G.; Costa, R.; Gugger, M.; Steinbeck, C.; Moreno, P.; Piel, J. Automated Structure Prediction of Trans-Acyltransferase Polyketide Synthase Products. *Nat Chem Biol* **2019**, *15* (8), 813–821. https://doi.org/10.1038/s41589-019-0313-7.

(6) Wagner, D. T.; Zhang, Z.; Meoded, R. A.; Cepeda, A. J.; Piel, J.; Keatinge-Clay, A. T. Structural and Functional Studies of a Pyran Synthase Domain from a *Trans* -Acyltransferase Assembly Line. *ACS Chem. Biol.* **2018**, *13* (4), 975–983. https://doi.org/10.1021/acschembio.8b00049.
